# Utilisation of Nanoparticle Technology in Cancer Chemoresistance

**DOI:** 10.1155/2012/265691

**Published:** 2012-11-14

**Authors:** Duncan Ayers, Alessandro Nasti

**Affiliations:** ^1^Department of Pathology, Faculty of Medicine & Surgery, University of Malta, Msida MSD 2060, Malta; ^2^School of Medicine, Kanazawa University Hospital, University of Kanazawa, Kanazawa 920-1192, Japan

## Abstract

The implementation of cytotoxic chemotherapeutic drugs in the fight against cancer has played an invariably essential role for minimizing the extent of tumour progression and/or metastases in the patient and thus allowing for longer event free survival periods following chemotherapy. However, such therapeutics are nonspecific and bring with them dose-dependent cumulative adverse effects which can severely exacerbate patient suffering. In addition, the emergence of innate and/or acquired chemoresistance to the exposed cytotoxic agents undoubtedly serves to thwart effective clinical efficacy of chemotherapy in the cancer patient. The advent of nanotechnology has led to the development of a myriad of nanoparticle-based strategies with the specific goal to overcome such therapeutic hurdles in multiple cancer conditions. This paper aims to provide a brief overview and recollection of all the latest advances in the last few years concerning the application of nanoparticle technology to enhance the safe and effective delivery of chemotherapeutic agents to the tumour site, together with providing possible solutions to circumvent cancer chemoresistance in the clinical setting.

## 1. Introduction

It is definitely not a matter of dispute that chemotherapy and its constituent cytotoxic agents play a vital role in the clinical management of the vast majority of cancer conditions. Chemotherapy measures focus on eradication of tumour presence or (at least) control the degree of tumour progression and metastasis. However, this therapy has its own critical flaws due to two major issues, namely, dose-dependent adverse conditions and the emergence of chemoresistance properties within the tumour.

## 2. Dose-Dependent Cumulative Adverse Effects

The issue of dose-dependent cumulative adverse effects derives from the pharmacological properties of cytotoxic chemotherapeutic agents, which are not tissue-specific and thus affect all tissues in a widespread manner. In addition, tissues having increased turnover rates, such as the gastro-intestinal system and skin, are more vulnerable to cytotoxic drug activity and are the most prevalent dose-limiting cumulative adverse effects in patients undergoing chemotherapy.  Table 1 describes in brief the pharmacology and adverse effects of a few of the most commonly prescribed chemotherapeutic agents that are implemented in many cancer chemotherapy strategies.

## 3. Tumour Chemoresistance Properties

**Figure 1:**
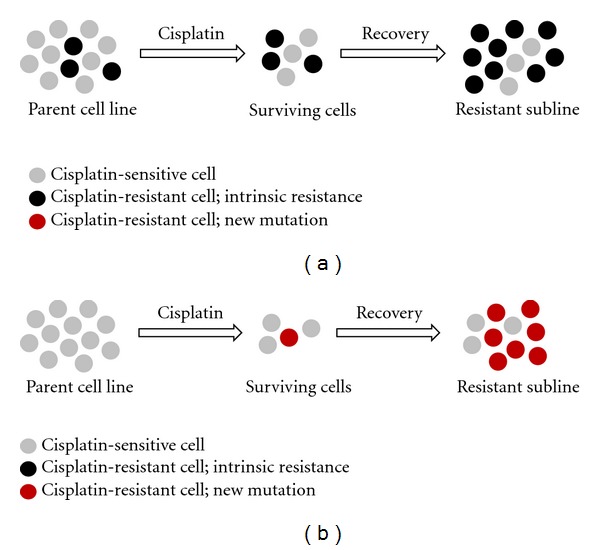
Overview of chemoresistance emergence, using cisplatin as an example for a conventional chemotherapeutic drug. Intrinsic chemoresistance (a) demonstrates the presence of tumour cell colonies that possess the optimal genetic and phenotypic characteristics to withstand exposure to cytotoxic agent activity. These characteristics were present in such cells prior to initial chemotherapy exposure and hence the term intrinsic chemoresistance. In acquired chemoresistance (b), the tumour cell line develops chemoresistance due to mutational driving forces following prolonged exposure to chemotherapeutic agents.

The emergence of chemoresistance within tumour cells of solid tissues is sadly one of the main reasons for treatment failure and relapse in patients suffering from metastatic cancer conditions [1]. Resistance of the tumour cell to chemotherapeutic agent exposure may be innate, whereby the genetic characteristics of the tumour cells are naturally resistant to chemotherapeutic drug exposure [2]. Alternatively, chemoresistance can be acquired through development of a drug resistant phenotype over a defined time period of exposure of the tumour cell to individual/multiple chemotherapy combinations [1, 2] (see Figure 1).

The biological routes by which the tumour cell is able to escape death by chemotherapy are numerous and complex. However, the major pathways enabling chemoresistance in cancer have been studied in detail and are summarised in Table 2.

## 4. Nanoparticle Technology

The introduction of nanotechnology in the last few decades has led to an undisputed boom in the conception and development of innovative methods for effective and safe delivery of small-molecule drugs and gene-based therapies to their intended target tissues. 

The advantages of exploiting nanoparticle delivery systems are many, such as the possibility to protect nuclease-labile drug therapies, such as short interfering RNAs (siRNAs) and microRNAs (miRNAs) during transit within the bloodstream [87, 88]. In addition, implementation of nanoparticle-based delivery systems has led to improved pharmacokinetic profiles for the specific drug being carried within such a system, together with enhanced targeting of the site of action of the drug [89–91]. The excellent review by Hu and Zhang [92] highlighted that nanoparticles also have the capacity to carry combination therapies of two drugs/small molecules and have demonstrated to be particularly effective in circumventing multidrug resistance (MDR) issues in multiple cancer models. 

**Figure 2:**
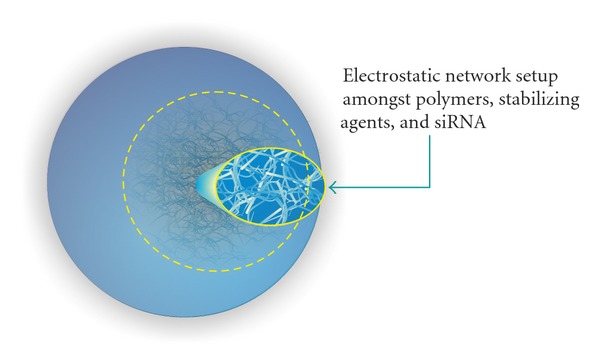
Representative example of a chitosan-based nanoparticle designed for the loading of individual siRNAs within the electrostatic network created by the nanoparticle internal infrastructure.

The chemical composition of nanoparticles, both from natural occurring compounds (see Figure 2) and synthetic ones (see Table 3), is varied and the selection of which nanoparticle to utilize for any individual drug delivery system is very much dependent on a multitude of factors such as the chemical nature of the drug to be transported, the loading capacity of the nanoparticle, and resultant pharmacokinetic and pharmacodynamics properties of the nanoparticle following drug loading [93].

**Figure 3:**
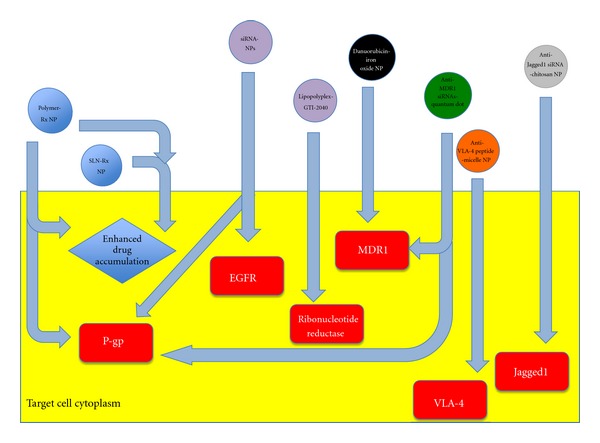
Visual representation of a selection of varying nanoparticle-based drug (Rx) delivery systems adopted for averting cancer chemoresistance properties. Polymer-based [70] and solid lipid nanoparticle-based [69] delivery systems (blue) allow for bypass of the drug efflux pump, acquired chemoresistance pathways and allow for enhanced drug accumulation within the target cell cytoplasm, together with P-gp downregulation [96]. RNA interference methods utilising short interfering RNAs (purple) have been incorporated in hydrogel nanoparticles for targeting of epidermal growth factor receptor, a key player in mediating cell adhesion methods of chemoresistance [71]. Another major MDR gene targeted by short interfering RNAs includes P-gp [97]. Lipopolycomplex nanoparticles were successful in enhancing the pharmacodynamic properties of the GTI-2040 oligonucleotide, targeting ribonucleotide reductase [98]. Ferromagnetic nanoparticles (black) have also been deployed for downregulation of the major chemoresistance gene MDR1 [72]. Micelle-based nanoparticles (orange) were found to be effective in delivering doxorubicin and VLA-4-specific peptides in multiple myeloma cells [76]. Quantum dots (green) containing siRNAs were also successfully deployed for downregulating MDR1 and P-gp expression in HeLa cell lines [81]. Chitosan nanoparticles (grey) incorporating Jagged1 siRNAs were also highly effective in circumventing MDR properties in taxane-resistant ovarian cell lines [99].

It is beyond the scope of this review to delve into the specific technical details regarding each individual type of nanoparticle utilized at present, as this has been already discussed extensively in other technical reviews and research articles within the literature [83, 84, 94, 95]. However, a brief summary encompassing the spectrum of varying nanoparticle compositions, key advantages together with toxicity profiles can be viewed in Table 3 and Figure 3.

## 5. Recent Advances in Nanoparticle-Based Cancer Chemoresistance Circumvention Methodologies

The study carried out by Kang et al. [69] demonstrated that administration of solid lipid nanoparticles containing doxorubicin (SLN-Dox) to the adriamycin-resistant breast cancer cell line MCF-7/ADR, which also overexpressed P-glycoprotein (P-gp), allowed for chemosensitisation of the cell line. This was induced due to enhanced accumulation of doxorubicin within the cell line, contributed by the nanoparticle-based delivery method, and thus the degree of apoptosis was enhanced [69]. 

The same principle of exploiting nanoparticle delivery to substantiate chemotherapeutic drug accumulation within the target cancer cell, with the ultimate goal of enhancing tumour chemosensitivity, was adopted in the study by Aryal et al. [70]. Polymer-cisplatin conjugate nanoparticles were developed and consequently delivered to A2780 human ovarian carcinoma cell line [70]. The added potential of this delivery system relied on the cisplatin analogue prodrug covalently linked to a poly(ethylene glycol)-based polymer, which only released its therapeutic payload in a low pH environment [70]. Consequently, clinical administration of such a delivery system would ensure that the drug will remain complexed whilst in transit within the bloodstream due to its neutral pH environment [70].

Additionally, RNAi therapeutics have come to rely much further on the utilization of nanoparticle delivery systems to exert their biological effects. The study by Dickerson et al. [71] elucidated the efficiency to knock-down genes such as epidermal growth factor receptor (EGFR) by the delivery of EGFR-specific siRNAs contained within core/shell hydrogel nanoparticles (nanogels). The nanogels were also coated with peptides targeting the EphA2 receptor to enhance delivery of anti-EGFR siRNAs within the targeted Hey tumour cells [71]. Consequently, the knock-down effect on EGFR led to enhanced chemosensitivity of cancer cells to taxane chemotherapy [71].

The implementation of nanoparticle technology has also demonstrated to aid the clinical effect of other therapies that were previously unsuccessful due to poor drug delivery issues. Jin et al. [98] developed transferrin conjugated pH-sensitive lipopolyplex nanoparticles with the capacity to bind specific oligodeoxynucleotides (GTI-2040 in this case). This delivery system allowed GTI-2040 to exert its effect on the R2 subunit of the chemoresistance factor ribonucleotide reductase in acute myeloid leukaemia cell line models [98]. The influence of ultilising such a delivery system was evident in that the 50% inhibitory concentration (IC(50)) for 1 *μ*M GTI-2040 decreased from 47.69 nM to 9.05 nM [98]. 

An additional nanoparticle delivery system, adopted against MDR in leukaemic conditions, was investigated by Cheng et al. [72]. This system combined magnetic iron oxide nanoparticles together with daunorubicin and 5-bromotetrandrin, which proved to possess a sustained release pharmacokinetic drug profile when administered to K562/A02 multidrug resistant leukaemic cell lines [72]. The principle behind the utilization of magnetic nanoparticles is due to the effects of magnetic field gradients positioned in a nonparallel manner with respect to flow direction within the tumour vasculature [73]. This allows for physical (magnetic) enhancement of the passive mechanisms implemented for the extravastation and accumulation of such magnetically responsive nanoparticles within the tumour microenvironment, followed by cellular uptake of the nanoparticles within the target tumour cell cytoplasm [73]. The magnetically responsive nanoparticle itself is composed of one or a combination of the three ferromagnetically active elements at physiological temperature, namely, iron, nickel, and cobalt [73]. The delivery system described by Cheng et al. [72] also aided in providing a dose-dependent antiproliferative effect on such cell lines, together with enhanced intracellular accumulation of daunorubicin and downregulated transcript expression of MDR1 gene, the main factor for induction of MDR in most cancer models [72]. These factors all contributed to a reduction in MDR and were directed by the level of endosomal-mediated cellular uptake properties of such nanoparticles [100].

In chronic myelogenous leukaemia (CML), a Bcr-Abl positive status induces MDR properties through multiple pathways, including resistance to p53 and Fas ligand-induced apoptotic pathways [101]. The delivery system devised by Singh et al. [101] consisted of magnetic nanoparticles combined with paclitaxel and was consequently administered to Bcr-Abl positive K562 leukaemic cell lines [101]. The addition of lectin functional groups to the nanoparticle complex served to aid cellular uptake by the target K562 cell line and also demonstrated a reduction in the IC(50) for paclitaxel within this cell line model [101].

Multiple myeloma is an additional tumour model that has seen benefit from the exploitation of nanoparticle technology in its therapeutic avenues [76]. The study by Kiziltepe et al. [76] succeeded in developing a micelle-based nanoparticle delivery system containing doxorubicin and very late antigen-4 (VLA-4) antagonist peptides [76]. This delivery method not only accomplished enhanced cytotoxic activity when compared to doxorubicin alone, but also the addition of VLA-4 antagonist peptides served well in circumventing the phenomenon of cell-adhesion-mediated drug resistance due to the resultant impaired VLA-4 mediated adhesion of multiple myeloma cells to the stroma of bone marrow within CB.17 SCID murine multiple myeloma xenograft models [76]. Additionally, drug accumulation within the stroma of the multiple myeloma murine xenograft models was also tenfold higher than the control murine model [76].

Yet another tumour model that has been investigated for the application of nanoparticle-based chemotherapy, for the purpose of avoidance of chemoresistance, is prostate cancer [102]. Gold nanoparticles are an attractive avenue for drug delivery researchers primarily due to their lack of complexity in their synthesis, characterization, and surface functionality [78]. Gold nanoparticles also have shape/size-dependent optoelectronic characteristics [78]. The endosomal-based route for gold nanoparticle cellular uptake can be viewed as the primary advantage for circumventing MDR within the tumour cell, since the drug efflux pump is bypassed and the nanoparticle-held chemotherapeutic agent is released within the acidic environment of the endosome and allowed to penetrate the tumour cell cytoplasm [79]. Consequently, tumour progression phenotypes such as cell proliferation and level of apoptosis are affected to direct an amelioration of patient prognosis.

Gold nanoparticle/antiandrogen conjugates were developed by Dreaden et al. [102], with the capacity to selectively bind to two surface receptors which are upregulated in prostate tumour cell surface. Thus allowing accumulation of the nanoparticle conjugate specifically within treatment-resistant prostate tumour cells [102]. Gold nanoparticles were also exploited in the study conducted by Tomuleasa et al. [103] for the purpose of reducing MDR hepatocellular carcinoma-derived cancer cells. The gold nanoparticles were loaded with doxorubicin, capecitabine, and cisplatin, followed by nanoparticle stabilization by L-aspartate [103]. The resultant cellular proliferation rates of the hepatocellular carcinoma cells treated with this nanoparticle-based therapy were found to be lowered drastically [103]. 

In the study carried out by Punfa et al. [104], the cytotoxic properties of curcumin on multidrug resistant cervical tumours were maximized through the development of a nanoparticle-curcumin drug delivery system. Curcumin was successfully entrapped within poly (DL-lactide-co-glycolide) (PLGA) nanoparticles, followed by the incorporation of the amino-terminal of anti-P-gp [104]. Consequently, the curcumin-nanoparticle conjugates were deployed onto the KB-V1 cervical cancer cell line, having upregulated P-gp expression, together with the KB-3-1 cell line that has a reduced P-gp expression level [104]. The results of this study demonstrated that nanoparticle conjugates bearing anti-P-gp surface markers were highly efficient in binding to the MDR-inducing surface protein, allowing enhanced cellular uptake and ultimately aid in the cytotoxic efficacy of curcumin due to increased accumulation of the drug, particularly within the KB-V1 cell line due to its exacerbated P-gp expression status [104]. 

Curcumin/doxorubicin-laden composite polymer nanoparticles were also developed in other studies [105] as a means of enhancing the pharmacokinetic and pharmacodynamics properties of curcumin, thus enhancing its MDR-modulating effect in the target tumour cells. The resultant nanoparticle complex was deployed onto several MDR tumour models such as acute leukaemia, multiple myeloma, and ovarian cancers, both *in vitro* and *in vivo* [105]. The results of this study highlighted the possibility of administration of lower doses of doxorubicin due to the circumvention of tumour MDR by efficient curcumin activity, thus enhancing the toxicity profile for doxorubicin in clinical use stemming from the reduction in cardiotoxicity and haematological toxicity dose-dependent adverse effects [105].

Retinoblastoma therapeutic avenues have also been increased due to the introduction of nanoparticle drug delivery technology. The study by Das and Sahoo demonstrated the effectiveness of utilising a nanoparticle delivery system which was dual loaded with curcumin together with nutlin-3a (which has been proven to stimulate the activity of the tumour suppressor protein p53) [106]. The results of this particular investigation highlighted an enhanced level of therapeutic efficacy on utilizing the nanoparticle-curcumin-nutlin-3a conjugates on the target retinoblastoma Y79 cell lines [106]. In addition, a downregulation of bcl2 and NF*κ*B was also observed following cell line exposure to the nanoparticle conjugates [106]. 

The nanoparticle-based drug delivery system designed by Saxena and Hussain [96] for its application against multidrug resistant breast tumours was novel in that the actual components of the nanoparticle biomaterials, namely, poloxamer 407 and D-*α*-tocopheryl polyethylene glycol 1000 succinate (TPGS), are both known to exert pharmacological activity against P-gp [96]. The drug utilized for nanoparticle loading in this case was gambogic acid, a naturally occurring cytotoxic agent though laden with issues of poor bioavailability and severe dose-limiting adverse effects [96]. Similarly to other studies mentioned above, the incorporation of a nanoparticle-based drug delivery system allowed for enhanced cellular uptake by the target breast cancer cell line MCF-7, thus leading to elevated drug accumulation on the intracellular level and ultimately inducing enhanced cytotoxic effects in the target breast cancer cell line [96].

A separate nanoparticle-based drug delivery system for use in circumventing MDR effects in breast cancer is the one developed by Li et al. [107]. In this study, the nanoparticle drug delivery system consisted of a dimethyldidodecylammonium bromide (DMAB)-modified poly(lactic-co-glycolic acid) (PLGA) nanoparticle core that was conjugated to doxorubicin, then consequently coated with a 1,2-dipalmitoyl-sn-glycero-3-phosphocholine (DPPC) shell [107]. This system has been described to be specifically effective against MCF-7 breast cancer cell lines overexpressing P-gp [107]. The results obtained from this particular study indicated an elevated accumulation of doxorubicin released from the nanoparticle complex, within the nuclei of the drug resistant MCF-7 cell line [107]. In comparison, the level of accumulation of freely administered (i.e., not utilising a nanoparticle-based drug delivery system) doxorubicin attained lower drug concentration levels within the same cell line [107]. Finally, the IC(50) levels for doxorubin on adriamycin-resistant MCF-7 have been observed to be lowered by 30-fold following the incorporation of this nanoparticle delivery system [107].

Apart from delivery of conventional chemotherapeutic drugs in drug resistant breast cancer cell line models, researchers also delved into the possibility of adopting siRNA therapeutic approaches, using the aid of nanoparticle drug delivery systems [97]. The study conducted by Navarro et al. [97] developed a nanoparticle-based delivery system for siRNAs targeting P-gp expression, with the nanoparticle constituent biomaterials being dioleoylphosphatidylethanolamine and polyethylenimine (PEI) [97]. Again, the reduction in P-gp expression led the path to enhanced cytoxic effects brought about by the exposure of the MCF-7 cell line to doxorubicin, thus this nanoparticle-siRNA therapy was successful in drastically reducing MDR in this cancer model [97].

Quantum dots have also been implemented as novel and effective drug delivery systems for circumventing multidrug resistance in cancer chemotherapy [81]. Researchers in this study developed a quantum dot-based drug delivery system that allowed anti-MDR1 siRNA and doxorubicin incorporation to two cadmium-selenium/zinc-selenium quantum dots that were eventually functionalized by *β*-cyclodextrin coupling to L-arginine or L-histamine [81]. Following deployment of these dual loaded quantum dots in the HeLa cervical cancer cell line model, elevated accumulation of doxorubicin within the tumour cells was denoted, together with a marked reduction in MDR1 and P-gp expression on analysis by reverse transcription real time quantitative polymerase chain reaction and western blotting [81]. In line with magnetic and gold nanoparticle platforms, quantum dots rely mainly on the endosomal method of tumour cellular uptake and therefore the drug efflux pump system is bypassed, with consequent reduction in MDR properties by the tumour cells [82]. Finally, the additional benefit of utilizing quantum dots as a drug delivery system is their capacity to be tracked in real time within specific areas of the target cells, due to their intrinsic fluorescence properties [81]. 

Apart from cell line studies, researchers have also looked into the feasibility of implementing nanoparticle-based drug delivery systems within *in vivo* models [108]. The study by Milane et al. [108] investigated the efficacy of utilising a EGFR-targeting polymer blend nanoparticles, loaded with paclitaxel and the mitochondrial hexokinase 2 inhibitor lonidamine. The nanoparticle polymer blend consisted of 70% polycaprolactone (PCL) incorporating a PLGA-polyethylene glycol-EGFR specific peptide that helped enable nanoparticle active targeting efficiency [108].

Following nanoparticle development, four groups of orthotopic MDR breast cancer murine models (MDA-MB-231 in nude mice) were treated with free paclitaxel, free lonidamine, free paclitaxel/lonidamine combination, or nanoparticle complexes containing paclitaxel/lonidamine combination [108]. The degree of toxicity of such treatments was also monitored through body weight change measurements, liver enzyme plasma levels, and white blood cell/platelet counts, together with H & E staining of tumour sections was carried out [108].

Tumour weight and other clinical parameters such as MDR protein marker (P-gp, Hypoxia Inducible factor *α*, Hexokinase 2, EGFR, Stem Cell factor) were observed over the course of 28 days after-treatment [108]. Following this 28-day period, the results demonstrated that only the murine model sample group exposed to the nanoparticle-based paclitaxel/lonidamine combination treatment was the only group to experience statistically significant tumour volume and density reduction, together with overall alteration of the MDR phenotype [108]. Toxicity effects due to paclitaxel and lonidamine were also drastically reduced when administered within the nanoparticle-based delivery system, which can ultimately provide enhanced tolerance by the cancer patient [108].

Other *in vivo* studies in this field include the investigations carried out by Shen et al. [109], which focused on the codelivery of paclitaxel and survivin short hairpin RNA (shRNA) for circumventing chemoresistance in lung cancer. The investigators utilized the pluronic block co-polymer P85 combined with D-*α*-Tocopheryl polyethylene glycol 1000 succinate (P85-PEI/TPGS) for developing the nanoparticles to be implemented in this study [109]. These nanoparticles were based upon triblock structural formation of hydrophilic poly(ethylene oxide) (PEO) blocks and hydrophobic poly(propylene oxide) (PPO) blocks, which also gives enhanced capacity to revert chemoresistance due to drug efflux pump inhibition properties, downregulation of ATPase activity and P85-induced inhibition of the gluthathione S-transferase compound detoxification enzyme at the subcellular level [109]. Paclitaxel and surviving shRNA were selected as the ideal drugs for nanoparticle delivery due to the former having poor efficacy due to chemoresistance within the tumour, and survivin was identified as highly expressed within chemoresistant tumours [109]. The *in vivo* activity of such nanoparticle systems (with/without paclitaxel and survivin shRNA) was evaluated on BALB/c nude mice injected with viable, paclitaxel-resistant, A549/T lung adenocarcinoma epithelial cells [109]. The results of this study demonstrated that deployment of the nanoparticle-based chemotherapeutic drug proved to have distinct enhancement of antitumour efficacy, when compared to deployment of the drug/s alone [109].

Chemoresistance to the aromatase inhibitor letrozole in postmenopausal breast cancer is another major therapeutic hurdle which was investigated *in vivo *[110]. Biodegradable PLGA-polyethylene glycol copolymer nanoparticles were developed by nanoprecipitation and designed to incorporate hyaluronic acid-bound letrozole (HA-Letr-NPs) [110]. The addition of hyaluronic acid served to enhance letrozole binding specificity to CD44 on the target tumour cell surface, with the expected consequences of enhanced drug accumulation within the target tumour cell cytoplasm and resultant re-sensitization of the target tumour cells to letrozole activity [110]. Such HA-Letr-NPs, once produced at a size of less than 100 nm diameter, were deployed within a letrozole-resistant murine xenograft tumour model [110]. The results of this study demonstrated a highly efficient nanoparticle-based drug delivery system, with the IC(50) for HA-Letr-NPs within the murine xenograft model being only 5 *μ*M when compared to the control groups, thus enhancing the *in vivo* aromatase enzyme activity within the xenograft and ultimately inducing a prolonged resensitising of the breast cancer tumour to letrozole activity [110].

The naturally occurring compound chitosan was also utilized for the development of *in vivo* nanoparticle-based therapies to circumvent ovarian cancer chemoresistance properties induced by overexpression of the Jagged1 notch ligand [99]. Murine orthotopic models, utilising female athymic nude mice, were injected with SKOV3Trip2 taxane-resistant ovarian cancer cell line and consequently, following one week, subjected to anti-Jagged1 siRNA/chitosan nanoparticle complexes (5 *μ*g dose of siRNA) with/without taxane, applied via intraperitoneal route twice weekly for a total period of five weeks [99]. The results of this study indicated that such nanoparticle-based complexes had the capacity to reduce tumour weight by over 70% within such murine models and also induced taxane sensitization within the tumour [99].

In a similar study, cationic liposome-polycation-DNA (LPD) and anionic liposome-polycation-DNA (LPD II) nanoparticle systems were developed to incorporate doxorubicin and VEGF siRNA within a murine ovarian cancer animal model [111]. Female, athymic nude mice were treated with 5 × 10^6^ cells of the MDR ovarian cancer cell line NCI/ADR-RES [111]. Once the murine tumours reached a size of approximately 16–25 mm^2^, the mice were consequently injected with individual nanoparticle complexes bearing either siRNA or doxorubicin at a dose of 1.2 mg/Kg in both cases, once daily for three consecutive days [111]. The results of this study demonstrated the effectiveness of such nanoparticle complexes for inhibiting tumour progression within the treated murine model groups, mainly due to impaired VEGF expression-related MDR [111].

Other human cancer conditions which were investigated for circumvention of tumour MDR properties through nanoparticle delivery include uterine sarcomas [112]. In the study carried out by Huang et al. [112], pH-sensitive mesoporous silica nanoparticles incorporating hydrazine and doxorubicin were developed for *in vivo* testing on murine models of doxorubicin-resistant uterine sarcoma. Since the composition of such nanoparticles specifically allow for cellular uptake through endocytosis, bypassing of the P-gp efflux pump induced a marked reduction in P-gp dependent MDR properties [112]. Consequently, the murine MDR tumour model treated with such nanoparticles demonstrated enhanced tumour apoptotic effects which were clearly confirmed by active caspase-3 immunohistochemical validation analysis [112].

## 6. Conclusion

The latest studies described above undoubtedly serve as a testament to the immense clinical value represented by nanoparticle technology. The ability of such nanoparticles, irrelevant of biomaterial composition to efficiently load individual or combinations of chemotherapeutic drugs and/or chemosensitising agents (such as curcumin) and novel RNA interference-based therapies has been clearly demonstrated above. This property provides an excellent escape mechanism for circumventing target tumour cell multidrug resistance properties based on drug efflux pump activity on the tumour cell surface, such as that exerted by P-gp. The overall advantage of deploying nanoparticles includes the drastic reduction in the IC(50) parameter for most of the carried chemotherapy agents, due to marked intracellular accumulation pharmacodynamics. This in turn would lead to a reduction in the clinical doses of the conventional cytotoxic agents required for chemotherapy, ultimately demonstrating a striking reduction in dose-dependent adverse effects in the oncology patient. 

Presently, this does not mean that nanotechnology-based translational therapies are not fraught with challenges, such as biocompatibility issues of the nanoparticle components and the level of complexity required for cost-effectively translating these novel therapies to the patient bedside. However, it is the firm belief of the authors that through constant accumulation of marginal gains in knowledge, derived from persistent and motivated researchers on a global scale, will ultimately overcome such scientific hurdles, thus nanoparticle-based drug delivery aided therapies will eventually become commonplace in the oncology clinic in the near future.

## Figures and Tables

**Table 1:** Overview of a selection of cytotoxic drugs commonly used in chemotherapy.

Cytotoxic drug	Mechanism of action	Major adverse effects	References
Cisplatin	Inter/intrastrand cross-link formation on nucleophilic N7 sites of adjacent adenine and guanine bases, leading to apoptosis.	Dose-dependent ototoxicity nephrotoxicity, neurotoxicity, and myelosuppression.	[3–9]

Carboplatin	Inter/intrastrand cross-link formation on nucleophilic N7 sites of adjacent adenine and guanine bases, leading to apoptosis.	Dose-dependent myelosuppression.	[3, 4]

Cyclophosphamide	Oxazaphosphorine DNA-alkylating pro-drug, activated by liver P450 cytochrome-induced 4-hydroxylation., thus forming DNA cross-linking phosphoramide mustard.	Neurotoxicity and nephrotoxicity due to chloroacetaldehyde formation by P450 cytochrome-induced oxidation.	[10]

Doxorubicin	Anthracycline-glucuronide conjugate prodrug activated by tumour *β*-glucuronidase, whereby the drug/DNA adduct possibly induces apoptosis by topoisomerase 2 inhibition or by a caspase cascade.	Dose-dependent cardiotoxicity, hepatotoxicity, and myelosuppression.	[11–15]

Etoposide	Topoisomerase II inhibitor, by raising the stability of the enzyme/DNA cleavage complex, ultimately leading to DNA strand breaks and apoptosis.	Possible secondary leukaemia due to chromosomal translocations induced by etoposide strand break activity, myelosuppression.	[16–22]

Ifosfamide (in severe NB cases)	Oxazaphosphorine DNA-alkylating prodrug, activated by liver P450 cytochrome-induced 4-hydroxylation, thus forming DNA cross-linking phosphoramide mustard.	Marked neurotoxicity and nephrotoxicity due to increased chloroacetaldehyde formation by P450 cytochrome-induced oxidation.	[10]

**Table 2:** Overview of methods adopted by tumour cells for acquiring chemoresistance properties.

Chemoresistance method	Description	Key player genes, proteins and/or signalling pathways	References
Drug efflux mechanisms	Utilisation of drug efflux active pump proteins for expulsion of multiple cytotoxics from tumour cell cytoplasm, thus inducing multidrug resistance (MDR).	ATP-dependent binding cassette (ABC) transporter proteins, multidrug resistance 1 (MDR1) gene, P-glycoprotein (P-gp), multidrug resistance 1 protein (MRP1), ABCG2.	[23–26]

Drug modulation	Tumour cell ability to inactivate, or at least attenuate, drug activation through the modulation of expression of key enzyme/s involved in the target cytotoxic drug's pharmacological and pharmacokinetic pathways.	Decreased expression or impairment of folylpoly-gamma glutamate-synthetase activity, resulting in antifolate drug resistance. Effect of glutathione on cisplatin inactivation-mediated chemoresistance.	[27–29]

Modification of drug targets	Upregulated expression or amplification of a target protein/enzyme, which may prove crucial for drug potency and effectiveness.	*β*-catenin, thymidylate synthase.	[30, 31]

Repair mechanisms following DNA damage	Exacerbated activity of components of the nucleotide excision repair pathway following tumour cell DNA damage.	Excision repair cross complementing 1 protein, microsatellite instability phenotype due to mutations in DNA mismatch repair genes.	[32–37]

DNA methylation mechanisms	Inhibition of key tumour suppressor genes leading to DNA methylations.	Caspase-8 promoter hypermethylation in neuroblastoma.	[38, 39]

p53 status	Dysfunction or loss of DNA damage/other stress induced p53 pathway-mediated apoptotic activity.	Mouse double minute 2 (Mdm2), p53 encoding gene (TP53).	[40–46]

Apoptotic pathway defects	Dysfunction or inactivation of the cytotoxic drug targeted intrinsic/extrinsic proapoptotic pathways in tumour cells.	Bcl-2 protein family, cellular FADD-like interleukin 1 beta converting enzyme-inhibitory protein (c-FLIP), cellular inhibitors of apoptosis proteins (cIAPs).	[47–59]

Proliferative pathway activation	Stimulation of cell proliferation through modulation of the PI3K and extracellular signal-regulated kinase (ERK) survival signalling pathways	Protein tyrosine kinases (PTKs) families, epidermal growth factor receptor (EGFR) family, transcription factor kappa B (NF*κ*B), Sirtuins (SIRTs).	[60–68]

**Table 3:** Overview of the major classes of nanoparticles utilised for chemotherapeutic drug delivery.

Nanoparticle (NP) composition	Unique characteristics and advantages	Adverse effects/toxicity of nanoparticle components	References
Solid lipid	Acidic pH of MDR tumour cells favours drug release from NP.	No haemolytic activity in human erythrocytes.	[69]

Polymer-based	Versatile acid-responsive drug release kinetics.	Minimal cytotoxicity observed on ovarian cancer cell lines.	[70]

Hydrogels	Easy synthesis, peptide-attachment facility for targeted delivery.	Nontoxic.	[71]

Magnetic (iron oxide)	Allows for physical (magnetic) enhancement of the passive mechanisms implemented for the extravastation and accumulation within the tumour microenvironment.	L-glutamic acid coated iron oxide nanoparticles demonstrated *in vitro* biocompatibility.	[72–74]

Micelle-based	Capable of solubilizing a wide range of water-insoluble drugs.	Relatively safe, though elevated doses can induce dose-dependent adverse effects such as hyperlipidaemia, hepatosplenomegaly, and gastrointentinal disorders.	[75–77]

Gold	Lack of complexity in their synthesis, characterization, and surface functionality. Gold nanoparticles also have shape/size-dependent optoelectronic characteristics.	Can induce cellular DNA damage.	[78–80]

Quantum dots	Capacity to be tracked in real time within specific areas of the target cells, due to their intrinsic fluorescence properties.	Potential long-term toxicity due to release of toxic components (e.g., Cadmium) and generation of reactive oxygen species.	[81, 82]

Chitosan	Naturally occurring compound, derived from crustacean shells.	High biocompatibility properties.	[83, 84]

Mesoporous silica	Physical characteristics (e.g., size, shape) can be easily modified to induce bespoke pharmacokinetic/pharmacodynamics profiles.	Possible membrane peroxidation, glutathione depletion, mitochondrial dysfunction, and/or DNA damage.	[85, 86]

## References

[1] Longley DB, Johnston PG (2005). Molecular mechanisms of drug resistance. *Journal of Pathology*.

[2] Kerbel RS, Kobayashi H, Graham CH (1994). Intrinsic or acquired drug resistance and metastasis: are they linked phenotypes?. *Journal of Cellular Biochemistry*.

[3] Goodsell DS (2006). The molecular perspective: cisplatin. *Oncologist*.

[4] Kaludjerović GN, Miljković D, Momcilović M (2005). Novel platinum(IV) complexes induce rapid tumor cell death in vitro. *International Journal of Cancer*.

[5] Berg AL, Spitzer JB, Garvin JH (1999). Ototoxic impact of cisplatin in pediatric oncology patients. *Laryngoscope*.

[6] Li Y, Womer RB, Silber JH (2004). Predicting cisplatin ototoxicity in children: the influence of age and the cumulative dose. *European Journal of Cancer*.

[7] Sastry J, Kellie SJ (2005). Severe neurotoxicity, ototoxicity and nephrotoxicity following high-dose cisplatin and amifostine. *Pediatric Hematology and Oncology*.

[8] Arany I, Safirstein RL (2003). Cisplatin nephrotoxicity. *Seminars in Nephrology*.

[9] Jiang M, Yi X, Hsu S, Wang CY, Dong Z (2004). Role of p53 in cisplatin-induced tubular cell apoptosis: dependence on p53 transcriptional activity. *American Journal of Physiology*.

[10] Chen C-S, Lin JT, Goss KA, He YA, Halpert JR, Waxman DJ (2004). Activation of the anticancer prodrugs cyclophosphamide and ifosfamide: identification of cytochrome P450 2B enzymes and site-specific mutants with improved enzyme kinetics. *Molecular Pharmacology*.

[11] Ateşşahin A, Türk G, Karahan I, Yilmaz S, Ceribaşi AO, Bulmuş O (2006). Lycopene prevents adriamycin-induced testicular toxicity in rats. *Fertility and Sterility*.

[12] Ferguson MJ, Ahmed FY, Cassidy J (2001). The role of pro-drug therapy in the treatment of cancer. *Drug Resistance Updates*.

[13] Swift LP, Rephaeli A, Nudelman A, Phillips DR, Cutts SM (2006). Doxorubicin-DNA adducts induce a non-topoisomerase II-mediated form of cell death. *Cancer Research*.

[14] http://www.chemocare.com/bio/doxorubicin.asp.

[15] Singal PK, Iliskovic N (1998). Doxorubicin-induced cardiomyopathy. *The New England Journal of Medicine*.

[16] Hande KR (1998). Etoposide: four decades of development of a topoisomerase II inhibitor. *European Journal of Cancer*.

[17] Duca M, Guianvarc’h D, Oussedik K (2006). Molecular basis of the targeting of topoisomerase II-mediated DNA cleavage by VP16 derivatives conjugated to triplex-forming oligonucleotides. *Nucleic Acids Research*.

[18] http://www.chemheritage.org/EducationalServices/pharm/chemo/readings/ages.htm.

[19] Burden DA, Kingma PS, Froelich-Ammon SJ (1996). Topoisomerase II-etoposide interactions direct the formation of drug- induced enzyme-DNA cleavage complexes. *Journal of Biological Chemistry*.

[20] Bagatell R, Rumcheva P, London WB (2005). Outcomes of children with intermediate-risk neuroblastoma after treatment stratified by MYCN status and tumor cell ploidy. *Journal of Clinical Oncology*.

[21] http://www.medicines.org.uk/EMC/.

[22] Mistry AR, Felix CA, Whitmarsh RJ (2005). DNA topoisomerase II in therapy-related acute promyelocytic leukemia. *The New England Journal of Medicine*.

[23] Robey RW, Massey PR, Amiri-Kordestani L, Bates SE (2010). ABC transporters: unvalidated therapeutic targets in cancer and the CNS. *Anti-Cancer Agents in Medicinal Chemistry*.

[24] Krishna R, Mayer LD (2000). Multidrug resistance (MDR) in cancerMechanisms, reversal using modulators of MDR and the role of MDR modulators in influencing the pharmacokinetics of anticancer drugs. *European Journal of Pharmaceutical Sciences*.

[25] Colone M, Calcabrini A, Toccacieli L (2008). The multidrug transporter P-glycoprotein: a mediator of melanoma invasion?. *Journal of Investigative Dermatology*.

[26] Norris MD, Bordow SB, Marshall GM, Haber PS, Cohn SL, Haber M (1996). Expression of the gene for multidrug-resistance-associated protein and outcome in patients with neuroblastoma. *The New England Journal of Medicine*.

[27] Assaraf YG (2007). Molecular basis of antifolate resistance. *Cancer and Metastasis Reviews*.

[28] Wilson TR, Longley DB, Johnston PG (2006). Chemoresistance in solid tumours. *Annals of Oncology*.

[29] Meijer C, Mulder NH, Timmer-Bosscha H, Sluiter WJ, Meersma GJ, De Vries EGE (1992). Relationship of cellular glutathione to the cytotoxicity and resistance of seven platinum compounds. *Cancer Research*.

[30] Yeung J, Esposito MT, Gandillet A (2010). *β*-catenin mediates the establishment and drug resistance of MLL leukemic stem cells. *Cancer Cell*.

[31] Copur S, Aiba K, Drake JC, Allegra CJ, Chu E (1995). Thymidylate synthase gene amplification in human colon cancer cell lines resistant to 5-fluorouracil. *Biochemical Pharmacology*.

[32] Bradbury PA, Kulke MH, Heist RS (2009). Cisplatin pharmacogenetics, DNA repair polymorphisms, and esophageal cancer outcomes. *Pharmacogenetics and Genomics*.

[33] Arora S, Kothandapani A, Tillison K, Kalman-Maltese V, Patrick SM (2010). Downregulation of XPF-ERCC1 enhances cisplatin efficacy in cancer cells. *DNA Repair*.

[34] Shen L, Issa J-PJ (2002). Epigenetics in colorectal cancer. *Current Opinion in Gastroenterology*.

[35] Kim H, An JY, Noh SH, Shin SK, Lee YC, Kim H (2011). High microsatellite instability predicts good prognosis in intestinal-type gastric cancers. *Journal of Gastroenterology and Hepatology*.

[36] Takahashi M, Koi M, Balaguer F, Boland CR, Goel A (2011). MSH3 mediates sensitization of colorectal cancer cells to cisplatin, oxaliplatin and a poly(ADP-ribose) polymerase inhibitor. *The Journal of Biological Chemistry*.

[37] Martin LP, Hamilton TC, Schilder RJ (2008). Platinum resistance: the role of DNA repair pathways. *Clinical Cancer Research*.

[38] Ren J, Singh BN, Huang Q (2011). DNA hypermethylation as a chemotherapy target. *Cellular Signalling*.

[39] Teitz T, Wei T, Valentine MB (2000). Caspase 8 is deleted or silenced preferentially in childhood neuroblastomas with amplification of MYCN. *Nature Medicine*.

[40] Maclaine NJ, Hupp TR (2011). How phosphorylation controls p53. *Cell Cycle*.

[41] Macchiarulo A, Giacchè N, Mancini F, Puxeddu E, Moretti F, Pellicciari R (2011). Alternative strategies for targeting mouse double minute 2 activity with small molecules: novel patents on the horizon?. *Expert Opinion on Therapeutic Patents*.

[42] Buchakjian MR, Kornbluth S (2010). The engine driving the ship: metabolic steering of cell proliferation and death. *Nature Reviews Molecular Cell Biology*.

[43] García-Escudero R, Martínez-Cruz AB, Santos M (2010). Gene expression profiling of mouse p53-deficient epidermal carcinoma defines molecular determinants of human cancer malignancy. *Molecular Cancer*.

[44] Mogi A, Kuwano H (2011). TP53 mutations in nonsmall cell lung cancer. *Journal of Biomedicine and Biotechnology*.

[45] Stilgenbauer S, Zenz T (2010). Understanding and managing ultra high-risk chronic lymphocytic leukemia. *Hematology*.

[46] Al-Ejeh F, Kumar R, Wiegmans A, Lakhani SR, Brown MP, Khanna KK (2010). Harnessing the complexity of DNA-damage response pathways to improve cancer treatment outcomes. *Oncogene*.

[47] Plati J, Bucur O, Khosravi-Far R (2011). Apoptotic cell signaling in cancer progression and therapy. *Integrative Biology*.

[48] Kushnareva Y, Newmeyer DD (2010). Bioenergetics and cell death. *Annals of the New York Academy of Sciences*.

[49] Allan LA, Clarke PR (2009). Apoptosis and autophagy: regulation of caspase-9 by phosphorylation. *FEBS Journal*.

[50] Rolland SG, Conradt B (2010). New role of the BCL2 family of proteins in the regulation of mitochondrial dynamics. *Current Opinion in Cell Biology*.

[51] Gandhi L, Camidge DR, de Oliveira MR (2011). Phase I study of navitoclax (ABT-263), a novel bcl-2 family inhibitor, in patients with small-cell lung cancer and other solid tumors. *Journal of Clinical Oncology*.

[52] Placzek WJ, Wei J, Kitada S, Zhai D, Reed JC, Pellecchia M (2010). A survey of the anti-apoptotic Bcl-2 subfamily expression in cancer types provides a platform to predict the efficacy of Bcl-2 antagonists in cancer therapy. *Cell Death and Disease*.

[53] Testa U (2010). TRAIL/TRAIL-R in hematologic malignancies. *Journal of Cellular Biochemistry*.

[54] Liu J, Fu XQ, Zhou W, Yu HG, Yu JP, Luo HS (2011). LY294002 potentiates the anti-cancer effect of oxaliplatin for gastric cancer via death receptor pathway. *World Journal of Gastroenterology*.

[55] Yu Z, Wang R, Xu L, Xie S, Dong J, Jing Y (2011). *β*-elemene piperazine derivatives induce apoptosis in human leukemia cells through downregulation of c-FLIP and Generation of ROS. *PLoS ONE*.

[56] Earnshaw WC, Martins LM, Kaufmann SH (1999). Mammalian caspases: structure, activation, substrates, and functions during apoptosis. *Annual Review of Biochemistry*.

[57] Petersen SL, Peyton M, Minna JD, Wang X (2010). Overcoming cancer cell resistance to Smac mimetic induced apoptosis by modulating cIAP-2 expression. *Proceedings of the National Academy of Sciences of the United States of America*.

[58] Lanuti P, Bertagnolo V, Pierdomenico L (2009). Enhancement of TRAIL cytotoxicity by AG-490 in human ALL cells is characterized by downregulation of cIAP-1 and cIAP-2 through inhibition of Jak2/Stat3. *Cell Research*.

[59] Gill C, Dowling C, O’Neill AJ, Watson RWG (2009). Effects of cIAP-1, cIAP-2 and XIAP triple knockdown on prostate cancer cell susceptibility to apoptosis, cell survival and proliferation. *Molecular Cancer*.

[60] Avraham R, Yarden Y (2011). Feedback regulation of EGFR signalling: decision making by early and delayed loops. *Nature Reviews Molecular Cell Biology*.

[61] Vidal F, de Araujo WM, Cruz ALS, Tanaka MN, Viola JPB, Morgado-Díaz JA (2011). Lithium reduces tumorigenic potential in response to EGF signaling in human colorectal cancer cells. *International Journal of Oncology*.

[62] Sheng Q, Liu J (2011). The therapeutic potential of targeting the EGFR family in epithelial ovarian cancer. *British Journal of Cancer*.

[63] Metro G, Finocchiaro G, Toschi L (2006). Epidermal growth factor receptor (EGFR) targeted therapies in non-small cell lung cancer (NSCLC). *Reviews on Recent Clinical Trials*.

[64] Al-Batran SE, Ruppert M, Jäger E (2011). Trastuzumab plus chemotherapy in gastric cancer overexpressing HER-2 and EGFR: a case report. *Onkologie*.

[65] Chuang SE, Yeh PY, Lu YS (2002). Basal levels and patterns of anticancer drug-induced activation of nuclear factor-*κ*B (NF-*κ*B), and its attenuation by tamoxifen, dexamethasone, and curcumin in carcinoma cells. *Biochemical Pharmacology*.

[66] Olmos Y, Brosens JJ, Lam EWF (2011). Interplay between SIRT proteins and tumour suppressor transcription factors in chemotherapeutic resistance of cancer. *Drug Resistance Updates*.

[67] Peck B, Chen CY, Ho KK (2010). SIRT inhibitors induce cell death and p53 acetylation through targeting both SIRT1 and SIRT2. *Molecular Cancer Therapeutics*.

[68] Lara E, Mai A, Calvanese V (2009). Salermide, a Sirtuin inhibitor with a strong cancer-specific proapoptotic effect. *Oncogene*.

[69] Kang KW, Chun MK, Kim O (2010). Doxorubicin-loaded solid lipid nanoparticles to overcome multidrug resistance in cancer therapy. *Nanomedicine*.

[70] Aryal S, Hu CMJ, Zhang L (2010). Polymer-cisplatin conjugate nanoparticles for acid-responsive drug delivery. *ACS Nano*.

[71] Dickerson EB, Blackburn WH, Smith MH, Kapa LB, Lyon LA, McDonald JF (2010). Chemosensitization of cancer cells by siRNA using targeted nanogel delivery. *BMC Cancer*.

[72] Cheng J, Wang J, Chen B (2011). A promising strategy for overcoming MDR in tumor by magnetic iron oxide nanoparticles co-loaded with daunorubicin and 5-bromotetrandrin. *International Journal of Nanomedicine*.

[73] Klostergaard J, Seeney CE (2012). Magnetic nanovectors for drug delivery. *Nanomedicine*.

[74] Zhang T, Qian L, Tang M (2012). Evaluation on cytotoxicity and genotoxicity of the L-glutamic acid coated iron oxide nanoparticles. *Journal of Nanoscience and Nanotechnology*.

[75] Torchilin VP (2007). Micellar nanocarriers: pharmaceutical perspectives. *Pharmaceutical Research*.

[76] Kiziltepe T, Ashley JD, Stefanick JF (2012). Rationally engineered nanoparticles target multiple myeloma cells, overcome cell-adhesion-mediated drug resistance, and show enhanced efficacy in vivo. *Blood Cancer Journal*.

[77] Lim SB, Banerjee A, Onyüksel H (2012). Improvement of drug safety by the use of lipid-based nanocarriers. *Journal of Controlled Release*.

[78] Arvizo RR, Bhattacharyya S, Kudgus RA, Giri K, Bhattacharya R, Mukherjee P (2012). Intrinsic therapeutic applications of noble metal nanoparticles: past, present and future. *Chemical Society Reviews*.

[79] Vigderman L, Zubarev ER Therapeutic platforms based on gold nanoparticles and their covalent conjugates with drug molecules.

[80] Di Guglielmo C, De Lapuente J, Porredon C, Ramos-López D, Sendra J, Borràs M (2012). In vitro safety toxicology data for evaluation of gold nanoparticles-chronic cytotoxicity, genotoxicity and uptake. *Journal of Nanoscience and Nanotechnology*.

[81] Li J-M, Wang Y-Y, Zhao M-X (2012). Multifunctional QD-based co-delivery of siRNA and doxorubicin to HeLa cells for reversal of multidrug resistance and real-time tracking. *Biomaterials*.

[82] Probst CE, Zrazhevskiy P, Bagalkot V, Gao X Quantum dots as a platform for nanoparticle drug delivery vehicle design.

[83] Zaki NM, Nasti A, Tirelli N (2011). Nanocarriers for cytoplasmic delivery: cellular uptake and intracellular fate of chitosan and hyaluronic acid-coated chitosan nanoparticles in a phagocytic cell model. *Macromolecular Bioscience*.

[84] Nasti A, Zaki NM, De Leonardis P (2009). Chitosan/TPP and chitosan/TPP-hyaluronic acid nanoparticles: systematic optimisation of the preparative process and preliminary biological evaluation. *Pharmaceutical Research*.

[85] Mamaeva V, Sahlgren C, Lindén M Mesoporous silica nanoparticles in medicine-Recent advances.

[86] Asefa T, Tao Z Biocompatibility of mesoporous silica nanoparticles.

[87] Alabi C, Vegas A, Anderson D (2012). Attacking the genome: emerging siRNA nanocarriers from concept to clinic. *Current Opinion in Pharmacology*.

[88] Howard KA (2009). Delivery of RNA interference therapeutics using polycation-based nanoparticles. *Advanced Drug Delivery Reviews*.

[89] Zhang L, Gu FX, Chan JM, Wang AZ, Langer RS, Farokhzad OC (2008). Nanoparticles in medicine: therapeutic applications and developments. *Clinical Pharmacology & Therapeutics*.

[90] Wang AZ, Gu F, Zhang L (2008). Biofunctionalized targeted nanoparticles for therapeutic applications. *Expert Opinion on Biological Therapy*.

[91] Hu C-MJ, Kaushal S, Cao HST (2010). Half-antibody functionalized lipid-polymer hybrid nanoparticles for targeted drug delivery to carcinoembryonic antigen presenting pancreatic cancer cells. *Molecular Pharmaceutics*.

[92] Hu C-MJ, Zhang L (2012). Nanoparticle-based combination therapy toward overcoming drug resistance in cancer. *Biochemical Pharmacology*.

[93] Shapira A, Livney YD, Broxterman HJ, Assaraf YG (2011). Nanomedicine for targeted cancer therapy: towards the overcoming of drug resistance. *Drug Resistance Updates*.

[94] Dufort S, Sancey L, Coll J-L (2012). Physico-chemical parameters that govern nanoparticles fate also dictate rules for their molecular evolution. *Advanced Drug Delivery Reviews*.

[95] Bitar A, Ahmad NM, Fessi H, Elaissari A (2012). Silica-based nanoparticles for biomedical applications. *Drug Discovery Today*.

[96] Saxena V, Hussain MD (2012). Poloxamer 407/TPGS mixed micelles for delivery of gambogic acid to breast and multidrug-resistant cancer. *International Journal of Nanomedicine*.

[97] Navarro G, Sawant RR, Biswas S (2012). P-glycoprotein silencing with siRNA delivered by DOPE-modified PEI overcomes doxorubicin resistance in breast cancer cells. *Nanomedicine*.

[98] Jin Y, Liu S, Yu B (2010). Targeted delivery of antisense oligodeoxynucleotide by transferrin conjugated pH-sensitive lipopolyplex nanoparticles: a novel oligonucleotide—based therapeutic strategy in acute myeloid leukemia. *Molecular Pharmaceutics*.

[99] Steg AD, Katre AA, Goodman B (2011). Targeting the notch ligand JAGGED1 in both tumor cells and stroma in ovarian cancer. *Clinical Cancer Research*.

[100] Osman O, Zanini LF, Frénéa-Robin M (2012). Monitoring the endocytosis of magnetic nanoparticles by cells using permanent micro-flux sources. *Biomed Microdevices*.

[101] Singh A, Dilnawaz F, Sahoo SK (2011). Long circulating lectin conjugated paclitaxel loaded magnetic nanoparticles: a new theranostic avenue for leukemia therapy. *PLoS ONE*.

[102] Dreaden EC, Gryder BE, Austin LA (2012). Antiandrogen gold nanoparticles dual-target and overcome treatment resistance in hormone-insensitive prostate cancer cells. *Bioconjugate chemistry*.

[103] Tomuleasa C, Soritau O, Orza A (2012). Gold nanoparticles conjugated with cisplatin/doxorubicin/capecitabine lower the chemoresistance of hepatocellular carcinoma-derived cancer cells. *Journal of Gastrointestinal and Liver Diseases*.

[104] Punfa W, Yodkeeree S, Pitchakarn P, Ampasavate C, Limtrakul P (2012). Enhancement of cellular uptake and cytotoxicity of curcumin-loaded PLGA nanoparticles by conjugation with anti-P-glycoprotein in drug resistance cancer cells. *Acta Pharmacologica Sinica*.

[105] Pramanik D, Campbell NR, Das S (2012). A composite polymer nanoparticle overcomes multidrug resistance and ameliorates doxorubicin-associated cardiomyopathy. *Oncotarget*.

[106] Das M, Sahoo SK (2012). Folate decorated dual drug loaded nanoparticle: role of curcumin in enhancing therapeutic potential of nutlin-3a by reversing multidrug resistance. *PLoS ONE*.

[107] Li B, Xu H, Li Z (2012). Bypassing multidrug resistance in human breast cancer cells with lipid/polymer particle assemblies. *International Journal of Nanomedicine*.

[108] Milane L, Duan Z, Amiji M (2011). Therapeutic efficacy and safety of paclitaxel/lonidamine loaded EGFR-targeted nanoparticles for the treatment of multi-drug resistant cancer. *PLoS ONE*.

[109] Shen J, Yin Q, Chen L, Zhang Z, Li Y (2012). Co-delivery of paclitaxel and survivin shRNA by pluronic P85-PEI/TPGS complex nanoparticles to overcome drug resistance in lung cancer. *Biomaterials*.

[110] Nair HB, Huffman S, Veerapaneni P (2011). Hyaluronic acid-bound letrozole nanoparticles restore sensitivity to letrozole-resistant xenograft tumors in mice. *Journal of Nanoscience and Nanotechnology*.

[111] Chen Y, Bathula SR, Li J, Huang L (2010). Multifunctional nanoparticles delivering small interfering RNA and doxorubicin overcome drug resistance in cancer. *Journal of Biological Chemistry*.

[112] Huang I-P, Sun S-P, Cheng SH (2011). Enhanced chemotherapy of cancer using pH-sensitive mesoporous silica nanoparticles to antagonize P-glycoprotein-mediated drug resistance. *Molecular Cancer Therapeutics*.

